# Supplementary material for: Are carbon emissions trading and green financial instruments synergistic? -Comprehensive quantitative research based on content analysis
Source: PLoS One. 2024 Mar 7;19(3):e0298601. doi: 10.1371/journal.pone.0298601 (PMC10919601; doi:10.1371/journal.pone.0298601)
Supplement: S1 Data — (ZIP) [file pone.0298601.s001.zip › Data/地级市Z_F工作报告.zip!/╔╜╢1⁄2/╘μ╫»╩╨/└┤╫╘╣1⁄2╓┌║┼í╛┬φ┐╦╩2╛▌═°í┐_2016.docx]

各位代表：

　　现在，我代表市人民政府向大会报告工作，请予审议，并请市政协各位委员提出意见。

一、“十二五”时期工作回顾

　　“十二五”时期，是我市经受住各种困难挑战，夺取改革发展新成就，为城市转型打下坚实基础的五年。全市上下在市委的正确领导下，主动适应经济发展新常态，千方百计抗下行稳增长、转方式调结构、抓改革惠民生，较好完成了“十二五”规划确定的主要目标任务。

　　（一）综合实力再上新台阶。2015年，预计全市生产总值完成2030亿元，公共财政预算收入完成149亿元，分别是2010年的1.5倍和2倍。社会消费品零售总额达到790亿元，是2010年的1.9倍。金融机构存款余额、贷款余额、中长期贷款余额，分别比“十一五”末增加542亿元、352亿元、188亿元。城镇、农村居民人均可支配收入分别达到25758元和12058元，年均增长10.9%和13.3%。五年间，固定资产投资累计完成6164亿元，是“十一五”的2.3倍。

　　（二）产业转型迈出新步伐。顺利通过国家资源枯竭城市转型中期评估，累计获得扶持资金64亿元。三次产业比例由8.6∶60.1∶31.3调整为7.5∶52.8∶39.7。工业转型升级取得积极进展，预计非煤产业增加值占比达到88.4%，比2010年提高17个百分点；联想化工、薛城能源、华润三九等项目竣工投产，水泥、轮胎等传统产业市场化重组成效明显，新能源、新医药、先进装备制造等新兴产业快速发展，高新技术产业产值占比达到19.8%。服务业发展步伐加快，投资年均增长14.5%，限上企业达到1476家，年交易额过亿元的专业市场发展到28家；创建为省乡村旅游示范市，台儿庄古城创建为5A级景区，4A级景区达到12个；成功举办中国非遗博览会、世界石榴大会、年博会等节会活动。现代农业发展走在全国前列，农机化综合作业水平达到83%，整建制创建为国家现代农业示范区。

　　（三）改革开放释放新活力。行政审批制度改革持续深化，“四清单一平台”运行体系基本建立，市级行政权力事项削减18.7%，非行政许可事项得到全面清理，激发了市场活力，市场主体达到27.6万家，是“十一五”末的1.7倍。市属工业企业改革改制基本完成，70家企业近4万名职工的遗留问题依法得到解决，市南工业区改革发展进入新阶段。财税体制改革有序展开，“全口径”政府预算体系全面建立，市级部门预决算和“三公”经费预决算全部公开，专项转移支付项目压减80%。金融改革取得突破，组建枣庄银行和枣庄农商银行，引进市外银行业金融机构4家。农村改革亮点纷呈，成功创建国家农村改革试验区，承包地“三权分置”等经验在全国推广。户籍制度改革破题，降低了农民进城落户“门槛”。招商引资和项目建设成效显著，累计落地外来投资过10亿元项目64个、世界500强企业项目10个；实施市级重点项目287个，完成投资1800多亿元，是“十一五”的2倍。去年进出口总额、出口额分别是2010年的1.8倍和1.9倍。引进培养“千人计划”、泰山学者等高层次人才12名。

　　（四）城乡面貌发生新变化。完善城乡规划体系，编制完成新型城镇化发展规划、城市总体规划和各区（市）控制性规划，以及中心城区道路网、整体风貌等专项规划。常住人口城镇化率达到53%，比“十一五”末提高5.7个百分点。城市基础设施累计投资180亿元，是“十一五”的1.5倍；新建改造城区道路210公里、各类地下管网535公里；供热面积达到2929万平方米，是“十一五”末的2倍。在新城，实施了总投资200多亿元的48个城建项目，市实验高中、实验小学、职业中专、市立医院新城分院、中医院、舜天购物中心、龙潭公园等建成启用，教育医疗、文体商贸体系基本形成。在老城，东西沙河治理工程全面竣工，解放路、胜利路等公铁立交建成通车，枣庄客运中心建成启用，中兴大道开工建设，整治背街小巷162条。抓好镇村建设，新型城镇化示范镇达到13个，100个村达到美丽乡村创建标准，城乡环卫一体化全覆盖通过省级认定。京沪高铁枣庄站、滕州东站和台儿庄复线船闸建成使用，枣临铁路、枣临高速公路竣工通车，枣庄港工程建设进展顺利；公路里程增加1408公里，BRT通车总里程全国最长。

　　（五）生态建设呈现新亮点。创建成为国家森林城市，建成环中心城区绿道200多公里，新增造林面积65万亩。全力打好水污染防治攻坚战，代表山东省参加迎淮核查，取得淮河流域和全国九大流域“双第一”。铁腕治理大气污染，全面完成建成区567台燃煤锅炉“煤改气”和6.3万辆黄标车淘汰任务，秸秆禁烧和治超治限工作走在全省前列。下大气力淘汰落后产能，关闭全部立窑水泥生产线和30万吨以下小煤矿，关停“小火电”机组8台，提前一年完成“十二五”节能目标，“一黑一灰”的煤城形象渐行渐远。狠抓生态修复，治理破损山体、废弃矿井矿坑、采矿塌陷地3万亩，关闭露天采石场82家、储煤场244个，新增省级以上湿地公园11个、森林公园5个。

　　（六）社会事业取得新进步。坚持每年办好一批惠民实事，累计民生支出710亿元。城镇新增就业28万人，农村劳动力转移就业34万人。企业退休人员养老金实现“十一连增”，城乡居民基本医疗保险、基本养老保险完成并轨，基础养老金实现翻番。新建改建中小学标准化校舍169万平方米、幼儿园29万平方米，校舍楼房率保持全省前列，学前三年入园率提高到84%；3个区（市）通过国家义务教育发展基本均衡县验收。扎实推进县级公立医院综合改革，改扩建医疗卫生设施38万平方米，每千人拥有医疗机构床位数比“十一五”末增加47%。新建市、区（市）福利中心5处、社区老年人日间照料中心160处、农村幸福院264处、农村社区服务中心360个。大运河中河台儿庄段列入世界遗产名录。信访维稳工作扎实有效，食品药品监管体系逐步完善，安全生产形势持续稳定。荣获全国双拥模范城和全省首批“四德”工程示范市称号。其他各项事业也都取得新进展。

　　（七）政府效能实现新提升。扎实开展党的群众路线教育实践活动和“三严三实”专题教育，政府系统作风持续好转。严格落实中央“八项规定”精神，持之以恒反对“四风”，清理违规公务用车675辆、办公用房2.7万平方米。自觉接受人大、政协监督，400件人大代表建议和2477件政协提案全部按时办结。完成新一轮政府机构改革，落实县以下机关公务员职务与职级并行制度。围绕建设法治政府，出台市政府工作规则和重大行政决策程序规定，建立政府法律顾问、新闻发布制度，依法行政水平不断提高。市长热线、市长信箱群众来电来信办结率达到96%。

　　各位代表，刚刚过去的2015年，面对持续低位运行的宏观经济形势，面对困难矛盾叠加的城市转型压力，我们咬定发展不放松，稳中求进，迎难而上，真抓实干，在转型发展道路上又迈出了坚实一步。主要做了以下六个方面的工作：

　　一是把抗下行稳增长作为首要任务。集中推出了加快工业提质转型、促进民营经济发展、招商引资和对外开放、财政金融支持实体经济的一揽子政策措施，打出了一套“组合拳”，有力支撑了经济发展的基本面。立足做大做强现有产业和企业，狠抓“265”产业集群培育工程，建立市级领导联系产业帮扶企业制度，积极为企业解决发展难题。深入推进与中泰证券、省农发行等16家大公司和省直单位的战略合作，为转型发展注入了强劲动力。深化政银企合作，新拨付市级接续还贷资金6.9亿元。2015年，预计全市生产总值增长7%，公共财政预算收入增长8.3%，固定资产投资增长13.9%，社会消费品零售总额增长10.7%，经济运行呈现“总体平稳、稳中有进、稳中向好”的态势。

　　二是把招商引资上项目作为关键抓手。务实精准开展招商，新开工外来固定资产投资亿元以上项目125个，其中10亿元以上项目12个。百个重点项目完成投资375亿元，红荷专用汽车、威智百科等32个项目竣工运营。服务业重点项目完成投资320亿元，真爱商城一期、万洲浙商城一期等20个项目建成运营，万达广场、奥特莱斯购物城、保利城市综合体等项目加快推进。实施农田水利基本建设工程255项。深入开展“外贸企业服务年”活动，进出口总额、出口额分别增长10.7%和21.6%，增幅居全省第3位和第1位。

　　三是把推进创新创业作为根本动力。加强创新平台建设，新增博士后工作站2家、省级工程实验室2家、省级企业技术中心9家。发明专利申请量、授权量分别增长60%和12%。工业技改投资完成360亿元，增长15%。枣庄创业大学挂牌运营，省、市级创业示范园发展到19家，SOHO珠江、鲁南网商谷等创业平台成为大众创业的沃土。发放创业担保贷款2.1亿元，扶持1700人成功创业。加快推进互联网小镇、鲁南数据中心建设，我市将成为省内继济南、青岛之后第三个宽带接入一级节点城市。创新农村合作金融模式，“枣庄经验”在全省推广。

　　四是把优化发展环境作为重要保障。统筹推进“六城同创”，着力打造宜居的生态环境和宜业的营商环境。创建成为国家园林城市，建成区新增绿地714公顷；全市主要河流水质稳定保持三类水标准，“蓝天白云、繁星闪烁”天数达到172天。全面启动国家卫生城市创建，顺利通过省级“除四害”先进城市达标验收。城区主要节点亮化工程扎实推进。建成启用中小企业公共服务平台，持续开展“庸懒散”专项整治、“百千百”民主评议和群众满意度调查，依法打击“三强五霸”，营商环境不断优化。

　　五是把办好惠民实事作为最大责任。民生支出占公共财政支出的比重提高到72.2%。市财政安排扶贫资金3800万元，5万人实现省标准脱贫。城乡居民基础养老金由每人每月75元提高到85元，基本医疗保险财政补助标准由每人每年320元提高到380元。北京中医药大学第四临床医学院正式运营，趟出了地校合作发展的路子。开工建设保障性安居工程1.1万套，改造农村危房1575户。基本完成农村公路安全生命防护工程。启动“气化枣庄”建设，70%的镇街步入“天然气时代”。文体中心体育馆建成启用，成功举办第三届全国智力运动会，提高了枣庄的美誉度。

　　六是把干在当下、成在实处作为鲜明导向。坚持一棒接着一棒跑、一锤接着一锤敲，一批群众期盼多年的项目终于开花结果。总投资30多亿元的庄里水库开工建设，同步规划了总投资70亿元的抽水蓄能电站项目；枣庄机场、城际铁路、新台高速纳入省综合交通网中长期发展规划，枣木高速东延、枣济线改建、沂台线南延、新城至滕州BRT等项目进展顺利，十电扩建、八一热电二期开工建设。丰源生物质发电、三维钢结构、天畅环保在“新三板”挂牌，雅百特成功借壳上市，实现了企业上市零的突破。枣庄高新区创建成为国家高新区，结束了我市没有国家级园区的历史。

　　各位代表！奋斗伴随艰辛，成绩来之不易。这得益于省委、省政府和市委的正确领导，得益于人大、政协和社会各界的监督支持，得益于全市人民团结一心、拼搏进取。在此，我代表市人民政府，向奋斗在各行各业的广大干部群众，向解放军、武警驻枣官兵和中央、省驻枣单位，向在我市创业发展的投资者、建设者，向所有关心、支持枣庄发展的港澳台同胞和海内外朋友，表示衷心的感谢和崇高的敬意！

　　同时，也要清醒地认识到，我市发展中还存在一些困难和问题：经济下行压力依然很大，部分行业和企业经营困难；经济总量仍然偏小，带动作用强的大企业、大项目较少；传统产业产能过剩，新兴产业支撑不强，服务业发展不快，结构调整任重道远；创新能力弱，开放程度低，转型升级、加快发展缺乏内生动力；城乡基础设施还有不少欠账，群众生产生活条件有待进一步改善；资源环境约束趋紧，节能减排压力较大，抓发展保重点与抓生态保环境的矛盾日益突出；脱贫攻坚任务繁重，教育、卫生等公共服务资源配置不均衡，生产安全、食药安全仍需加强；一些政府工作人员存在不作为、慢作为、乱作为现象，作风建设需要常抓不懈，等等。我们一定坚持问题导向，勇于担当，精于进取，成于实干，决不让矛盾累积，决不让困难阻挡，用看得见摸得着的发展业绩，回报全市人民的信任和重托！

二、“十三五”时期发展的目标要求

　　“十三五”时期，是全面建成小康社会的决胜期，是我市加快新旧动能转换、实现城市转型的攻坚期。一方面，国际金融危机的深层次影响依然存在，国内结构性改革带来的阵痛仍将持续，各种矛盾愈加凸显，各种挑战前所未有。另一方面，世界新一轮科技革命蓬勃兴起，国家全面深化改革持续发力，山东西部经济隆起带建设纵深推进，我市交通区位优势、生态环境优势、政策叠加优势集中显现，广大干部群众盼发展、谋发展、促发展的热情空前高涨，有利于我们坚定赶超发展的信心和决心，在新起点上创造新的业绩。

　　今后五年，政府工作的指导思想是：深入贯彻落实党的十八大、十八届三中四中五中全会和习近平总书记系列重要讲话精神，按照中央“四个全面”战略布局和省委“一个定位、三个提升”要求，坚持创新、协调、绿色、开放、共享发展理念，着力深化供给侧结构性改革，紧紧围绕“在全面建成小康社会进程中走在前列”这一目标，牢牢把握转型升级和经济文化融合发展“两大高地”战略定位，着力推动创新、开放、生态“三大跨越”，加快构建现代产业、新型城镇、社会治理和民生保障“四个体系”，干在当下，成在实处，努力打造自然生态、宜居宜业的新枣庄。

　　今后五年，我们的奋斗目标是：经济保持中高速增长，在提高发展平衡性、包容性、可持续性的基础上，提前实现经济总量和居民人均收入比2010年翻一番，比全国提前两年实现贫困人口全面脱贫，与全省同步全面建成小康社会。实现上述目标，必须在六个方面作出不懈努力：

　　（一）统筹推进三次产业融合转型发展。坚持把推动产业转型、发展实体经济作为城市转型的战略支点，围绕去产能、去库存、去杠杆、降成本、补短板“五大任务”，推进供给侧结构性改革，增加有效供给，培育消费热点，努力构建结构合理、特色鲜明的产业发展新格局。推动工业提档升级，搭乘中国制造2025班车，以“265”产业集群培育为带动，推动生产方式向柔性、智能、精细转变，努力做大新型化工、装备制造、新能源、新医药、新型建材、电子信息、食品加工、橡胶轮胎、纺织服装、节能环保等十大产业基地，把“工业强市”的梦想转化为“枣庄制造”的强大力量；非煤产业增加值占比达到90%以上。推动服务业提速做优，顺应消费个性化、多样化趋势，推动生产性服务业向价值链高端延伸，生活性服务业向高品质转变，做大生态旅游、特色文化、商贸市场、现代物流、健康养生、体育产业、金融保险、电子商务等八大业态；服务业增加值占比达到45%以上。推动农业提质增效，围绕国家农村改革试验区和现代农业示范区建设，加快转变农业发展方式，提高农业产业化、标准化、品牌化、高效化水平；在全省率先基本实现农业现代化。

　　（二）统筹推进智力资源协同创新发展。把创新作为助力经济发展的新引擎，激活各类创新要素，让全社会拥抱“创时代”。加强企业创新能力建设，完善科技创新政策扶持体系，鼓励企业引进、消化、攻克更多关键核心技术，抢占行业发展先机；新增高新技术企业100家，高新技术产业产值占比达到24%；工业技改投入年均增长15%以上。加强“双创”载体建设，推广新型孵化模式，发展众创、众包、众扶、众筹空间，优化资源配置、凝聚大众智慧，让创新创业在枣庄蔚然成风；各级各类孵化器达到20家，在孵企业超过1000家。加强智慧枣庄建设，加快推进网络基础设施建设，放大宽带接入一级节点效应，更好地对接“信息高速公路”，形成若干互联网经济产业园和IT企业“服务区”，推动互联网与城市建设、产业发展、群众生活深度融合。

　　（三）统筹推进对内对外包容开放发展。积极融入国家开放大格局，主动参与“一带一路”建设，构建互利共赢、多元平衡、安全高效的开放型经济新体系。提升“引进来”的质量，持之以恒抓好招商引资、招才引智，集中力量招引一批能够带动产业转型的大项目、好项目；鼓励扩大消费品进口，有选择地进口能源资源性产品、关键设备和重要零部件；发挥高铁的“磁场效应”，吸引高层次人才和研发团队向枣庄集聚，引进100名创新创业领军人才。增强“走出去”的能力，推动重点行业积极参与全球产业链布局，提升高附加值产品出口比重，让更多的“枣庄产品”走向国际市场。做优“留得住”的环境，创新人才激励机制，注重培养本土优秀人才，支持企业家俱乐部建设，加大实用型技能人才培训力度；每年至少培养1000名“金蓝领”。

　　（四）统筹推进生产生活低碳绿色发展。把生态作为最大的发展优势，全力打造生态枣庄。推进绿色治理工程，深入开展大气污染防治行动，让群众经常看得到日月星辰、闻得到鸟语花香；加强流域污染治理，实行从水源地到水龙头的全过程监管，保障主要河流断面水质稳定达标和群众饮水安全。推行绿色生产模式，运用新设备、新技术改造传统产业，加快发展绿色低碳产业；积极推进园区循环化改造，打造循环经济产业链；主要污染物排放总量、万元生产总值能耗完成省下达任务。构筑绿色安全屏障，提升国家森林城市和园林城市建设水平，推进海绵城市建设，实施中心城区山体水系治理工程，把好山好水好风光融入城市，塑造“青山拥城、绿道环城、碧水绕城”的城市形象。健全绿色发展机制，落实主体功能区规划，完善生态补偿制度，严格排污费征收，对重点生态功能区、重点水源保护区加大转移支付力度；建成国家卫生城市、生态园林城市和省级生态市。

　　（五）统筹推进区域城乡一体协调发展。坚持以人为本，顺应城乡发展规律，统筹规划、建设、管理“三大环节”，统筹空间、规模、产业“三大结构”，统筹生产、生活、生态“三大布局”，建设和谐宜居、富有活力的新型城镇化体系；户籍人口城镇化率年均提高2个百分点。进一步优化发展格局，突出组团式城市特色和大新城引领作用，推行多规合一，以中心城区为核心、区（市）城区为支撑、示范镇为节点、农村新型社区为依托，构建“四位一体”新型城镇化格局；中心城区按照“西城扩容、东城提质、一体发展”的思路，加强公共基础设施建设，实现设施共建共享、产业互补互动、要素集中集约。进一步强化基础支撑，推进快速铁路、高速公路、民航机场、沿运港口等重大交通基础设施建设，形成高效便捷、水陆空交织的立体交通网络；构建京台、枣临、新台、枣木及东延高速公路大回环，建成全省第二个高速绕城的城市；大力实施水网、气网、热网、电网、公交网改造提升工程，加快城市地下综合管廊建设，厚植城市发展优势。进一步加强管理服务，建立统一规范的数字化管理平台，推行城市网格化、精细化管理，促进城市管理现代化；加快公共服务向镇村延伸，有序推进农村人口就地市民化；深入开展城乡环境综合整治，全面完成农村存量危房和厕所改造任务；稳步推进不动产统一登记；扎实开展文明城市创建，深化“四德”工程建设，让争做“枣庄好人”成为时代风尚。

　　（六）统筹推进社会民生共建共享发展。坚持人人参与、人人尽力、人人享有的原则，强化政策兜底，体现公平正义，增进民生福祉。推动各类教育均衡协调发展，加快基础教育综合改革，优化教育资源布局，多渠道解决“大班额”问题，推动教育内涵式发展；普及高中阶段教育，大力发展职业教育，劳动年龄人口受教育年限提高到15年；家庭困难学生资助实现全覆盖。打造健康枣庄，加强医疗基础设施建设，强化公共卫生服务，建成覆盖城乡的基本医疗卫生制度；深化“食安枣庄”建设，推进全民健身计划，提升群众健康水平。繁荣公共文化，构建覆盖全域的公共文化服务体系，推进文化事业与文化产业协调发展，增强文化软实力。促进社会和谐，坚决打赢脱贫攻坚战，决不让一个人在全面建成小康社会的进程中掉队；完善社保和救助体系，实现基本社会保险城乡全覆盖；搭建就业平台，拓宽就业渠道，让更多的人“有活干、有钱挣、有盼头”；坚持全面依法治市，推进“平安枣庄”“法治枣庄”建设，维护社会和谐稳定。

　　我们坚信，只要全市上下凝聚起“枣庄发展人人有责”的共识，万众一心、发奋图强，脚踏实地、久久为功，就一定能把美好蓝图变为生动现实！

三、2016年政府工作目标任务

　　2016年是“十三五”的开局之年，做好今年的工作至关重要。今年经济社会发展的主要预期目标是：全市生产总值增长7.5%左右，固定资产投资增长13%左右，规模以上工业增加值增长7%以上，公共财政预算收入增长7%以上，社会消费品零售总额增长11%左右，进出口总额增长6%，城镇、农村居民人均可支配收入分别增长8%和9%，居民消费价格涨幅控制在2.5%左右，城镇登记失业率控制在3.8%以内，全面完成省下达的年度节能减排约束性指标。重点抓好七个方面的工作：

　　（一）突出抓好产业支撑。立足现有产业基础，加快结构调整，做强主导产业、做优特色产业、做大新兴产业，构建现代产业体系，提高供给侧的质量和效益。

　　一是着力发展先进制造业。编制实施重点产业转型升级计划，大力推进“265”产业集群发展。拉长衍生一批，开展化工产业三年提升行动，加快瑞达聚丁烯、JFE苯酐、建阳炭黑等项目建设，推进煤气化向烯烃下游衍生物、醋酸纤维方向发展，煤焦化向苯加氢、电子级化学品方向发展，抢占煤基新材料产业制高点。推动鲁南机床、威达重工加强与国内外行业领军企业的战略合作，促进装备制造业上游向精密铸造发展，下游向智能化、高端化发展。技改提升一批，抓好十电扩建、丰源轮胎、青纺联和联润精纺、恒宇和远通纸业等技改项目，降低企业生产成本，实现提质增效。培植壮大一批，抓好三九、海王、威智、康德莱等项目，推进鲁南医疗器械产业园建设，膨胀医药产业规模。加快国开、华电、亿兆等新能源项目建设，力争年内光伏并网发电能力达到300兆瓦。抓好中材科技、丰源森木定向板、东方光源光纤预制棒等项目，推动新材料、节能环保等产业加速发展。兼并重组一批，支持煤炭、玻璃等传统产业引入战略合作伙伴，对内整合、对外重组、抱团发展，增强市场竞争力和抗风险能力。引导中小企业通过市场化并购重组“找好婆家”“靠大树”，实现借力借势发展。关停淘汰一批，继续实施工业绿动力计划，严格项目审核管理，限制新增过剩产能，加大环保、能耗等指标约束，再淘汰一批火电、焦炭、印染等行业的落后产能。

　　二是着力发展现代服务业。发展商贸市场，抓好万达广场、银座鲁南旗舰店、中央广场、民泰同购、兴盟国际等项目，努力打造功能完善、布局合理、时尚现代的商贸市场体系。发展现代物流业，借助交通区位优势，培育高铁经济、沿运经济，抓好鲁南铁水联运物流园、峄州港和台儿庄港仓储物流产业园等项目，促进我市与高铁、运河沿线城市经济互联、业态互补。发展旅游产业，支持微山湖红荷湿地、抱犊崮·熊耳山、冠世榴园创建5A级景区，开展特色旅游村镇和乡村旅游示范单位创建，推进区域旅游合作，塑造“枣庄旅游”品牌。发展文化产业，深入挖掘和弘扬地方优秀传统文化，抓好台儿庄国家级文化产业园、中兴矿山公园、铁道游击队主题公园、天穹影视基地等项目，提升“枣庄文化”影响力。发展健康养生业，鼓励医疗机构和其他社会资本兴办健康养生产业，抓好市社会福利（养老）服务中心二期、市残疾人康复中心、福源养生园等项目建设，促进医养结合。发展金融服务业，支持枣庄银行、枣庄农商银行立足“强基固本”加快发展，积极引进异地股份制商业银行和证券、基金、保险等机构，大力发展普惠金融和融资租赁。创新银保合作模式，拓展“信贷+保险”业务。加快要素市场建设，培育知识产权评估、检验检测、会计审计等生产性服务业。

　　三是着力发展现代农业。抓好国家现代农业示范区建设，努力走出独具特色的“枣庄农业”发展之路。培育新型职业农民，大力开展农业技术、经营管理、科技创新等培训，年内培训新型职业农民2000人。构建新型农业经营体系，培育壮大新型经营主体，新发展家庭农场、农民合作社500家，农地适度规模经营比例提高到52%。做大石榴、马铃薯、大枣等特色产业，拉长农产品加工转化链条，培植壮大穆拉德、祥和、春藤、莺歌等10大农业龙头企业，扶持10家小型农业企业“规下转规上”。全面落实粮食安全责任，加强粮食生产、储备、流通能力建设。强化新型物质技术装备，落实藏粮于地、藏粮于技战略，加强耕地保护，新增高标准农田18万亩。开展“农业科技展翅”行动，发展大型复式农业机械，推广立体种养、生物共养、休耕轮作等生产模式。健全新型农产品质量管理体系，完善市、区（市）、镇街三级农产品质量安全监管网络，加快农产品质量安全追溯体系建设，打造从田间到餐桌的食品安全链条。

　　四是着力发展互联网产业。以“智慧枣庄”为引领，抓好鲁南数据中心建设，搭建“枣庄云平台”，推动信息技术与产业融合发展，为实体经济插上互联网的翅膀，让互联网扎根于实体经济的土壤。实施“制造业＋互联网”行动，深入推进“两化融合”，鼓励企业推行“机器换人”，推广智能制造、柔性生产、个性化定制、众包设计等新模式，促进“枣庄制造”向“枣庄智造”转变。实施质量强市、品牌兴市战略，深入开展枣庄工业品线上行动，用好“好品山东”等电商平台，提升企业网络营销能力和品牌价值。实施“服务业＋互联网”行动，加快互联网小镇建设，打造互联网经济产业园。推动智慧景区建设，发展在线旅游。抓好枣庄电子商务产业园、贵诚e购网商平台等载体建设，支持网商网店销售“枣庄产品”。实施“农业＋互联网”行动，开展农业物联网示范应用建设试点，搭建农业云服务、农技推广平台，提高农业智能化水平。加强与阿里巴巴等企业的合作，培育发展棠棣峪、大马湾等一批电商“萌芽村”，让枣庄的优质农产品走出深山、走进市场。

　　（二）突出抓好对外开放。牢固树立大开放促进大发展的理念，凝神聚力招商引资上项目，稳住投资这驾马车；千方百计融入国际市场，做大出口这驾马车。

　　一是在引资引智上跨进一步。更加注重产业招商，围绕做大做强特色优势产业，着眼于建链、补链、强链，谋划一批有潜力、见效快的产业项目，深化与各地商会、中介机构的合作，搞好包装推介、合作共建。更加注重招大引强，发挥企业主体作用，面向世界和中国500强企业、行业领军企业，聚焦京沪江浙、港台日韩等重点区域，集中力量引进一批拥有核心技术、占据价值链高端的大项目、好项目。力争年内新开工过亿元项目130个以上。更加注重招才引智，编制急需紧缺专业人才目录，引进一批高精尖技术人才、专业管理人才和团队。加强与浙江大学、北京中医药大学等知名院校的合作，争取在我市设立工业技术研究院和教学科研机构，带动更多高水平专家院士走进枣庄、服务枣庄。组织本地企业与高等院校、科研院所、高新技术企业对接，引进转化一批先进技术，破解产业发展瓶颈。

　　二是在园区建设上跨进一步。创新园区规划建设管理模式，积极构建以国家级园区为引领、省级开发区为支撑、特色产业园为补充的“雁阵式”园区发展格局。强化规划引导，绘制全市“产业地图”，对枣庄高新区、省级开发区统一进行“二次规划”，支持项目飞地建设，引导园区差异定位、互补发展。强化基础建设，引导社会资本开发工业地产，建设标准化厂房、仓储和生活设施，完善园区“九通一平”配套，提升承载能力。强化要素保障，开展园区土地清理整治行动，坚决清理“空壳”企业、“僵尸”企业，盘活利用闲置土地、厂房、设备，实现“腾笼换鸟”、二次创业。探索“园区+担保+银行”模式，积极开展企业无形资产质押，以及生产设备、产成品等动产抵押。强化体制创新，探索“园镇合一”管理模式，赋予园区更充分的管理权限，逐步实现人权、财权、事权相匹配。支持枣庄高新区创新体制、争先进位、跨越发展，支持滕州经济开发区争创国家级开发区。

　　三是在外经外贸上跨进一步。多措并举“扩规模”，开展“国际市场开拓年”活动和零业绩企业“唤醒行动”，帮促新备案企业，引进生产型外贸企业，培植新的外贸增长点。加大出口扶持力度，支持企业参加境内外展会、承包境外工程、出口成套设备、输出高端劳务。持之以恒“提质量”，强化自主出口品牌建设，提升纺织服装、机械机床、化工建材、农副产品等出口商品质量，支持企业参加国际产能转移经贸活动，通过出口和对外投资去产能。搭建平台“优服务”，设立外贸综合服务中心，引进、培植国际贸易综合服务企业，规划建设外贸物流基地，大力发展跨境电商业务。发挥好海峡两岸交流基地作用，深化对台经贸文化合作。抓好培训“强人才”，选择成长性好的企业，实施外经贸业务中长期培训计划，培养企业外贸管理和实际操作人才，建设一支开拓意识强、业务素质高的外贸人才队伍。

　　（三）突出抓好重点改革。更加注重改革的系统性、整体性、协同性，环环相扣，循序渐进，使改革成为转型发展的不竭动力。

　　一是深化行政审批制度改革。高效运行“四清单一平台”行政权力体系，持续推进简政放权、放管结合、优化服务。推行区域化评估评审，严格执行涉审中介服务收费目录，依法依规清理投资项目审批前置事项，优化审批流程，促进项目审批“再提速”。凡国家、省明令取消或禁止的事项，一律不准变相设置审批门槛；凡市场能够自我调节，公民、法人和其他组织能够自主决定、自担风险、自律管理的事项，政府一律不再干预；凡能够通过事中事后监管达到管理目标的事项，一律取消事前审批，加快形成边界清晰、分工合理、权责一致、运转高效的政府职能体系，着力打造亲商、爱商、安商的营商环境。

　　二是深化国企国资改革。坚持因地制宜、一企一策，宜独则独、宜控则控、宜参则参，大力推进国有企业产权多元化。健全法人治理结构，推动市属国有企业建立规范化公司制度。引进央企、省企、外企和大型民企，参与我市国企改制重组和国有资本投资项目建设运营，稳步发展混合所有制经济。试点建立国有企业职业经理人制度，实施市属国有企业负责人薪酬制度改革，探索企业员工持股办法。抓好国有资本投资运营公司改组试点，促进经营性国有资产集中统一监管。

　　三是深化农村改革。继续按照“三延伸一创新”的思路，加快国家农村改革试验区建设，拓宽“三农”发展空间。抓好农村资产产权确权登记，强力推进农村集体建设用地、宅基地和农民房屋确权，赋予农民更多的财产权，真正让农民吃上“定心丸”。实现农村集体产权股份制改革试点镇街全覆盖，保障村集体和农民的合法权益。完善镇级农村产权交易机构服务功能，让农村资产“活”起来。规范合作经营行为，保障农地农用。扩大农业保险覆盖面，抓好马铃薯、生猪目标价格保险试点，减少农产品价格波动给农民带来的损失。抓好新型农村合作金融试点提质扩面，探索融资担保基金等新型互助模式，创造更多的“枣庄经验”。

　　四是深化财税体制改革。完善全面规范、公开透明的预算管理体系，实行中期财政规划管理，建立跨年度预算平衡机制。深化国地税征管体制改革，实行合作办税、综合治税，全面推开营改增改革，培植壮大可持续财源。科学划分市、区（市）的事权和支出责任，完善一般性转移支付增长机制。整合盘活机关事业单位闲置房产、土地等资源，唤醒更多沉睡资产融入资本要素市场。加强政府性债务管理，规范各类融资行为，坚决守住不发生系统性和区域性风险的底线。

　　五是深化投融资体制改革。搭建更多的政府投融资平台，推进资产资本化、资本债券化。发挥鲁南投资、金色城乡、恒通管廊等平台的作用，筹集更多资金用于城乡建设。加强市、区（市）融资担保体系建设，积极运作国开行、农发行贷款项目，争取更多的政策性资金和地方债券额度。参股省PPP项目基金，加大PPP项目运作力度，拓宽融资渠道。引入高水平团队管理运营好股权投资引导基金，市场化方式助推产业发展。充分利用多层次资本市场，推动股权众筹融资试点，引导企业在各类股权交易市场挂牌，支持丰元化学、泰和水处理等企业上市。

　　（四）突出抓好创新创业。深入实施创新驱动发展战略，完善政策扶持体系，点燃创新创业的星星之火，让更多的人梦想成真。

　　一是提升创新创业能力。加大产学研合作力度，鼓励企业开发新产品、新技术、新工艺，形成更多核心技术和优势项目。承担省级以上创新项目100项。发挥市产业技术协同创新中心的作用，推行科技创新券制度，推进科技成果产业化。实施科技“小巨人”企业培育工程和“小升高”助推计划，帮促20家小微企业成长为高新技术企业。设立专项资金，培训100名企业家、1000名中小微企业经营管理人员和首席信息官。抓好岗前职业技能和创业培训，提高创业成功率。

　　二是搭建创新创业平台。支持企业申报技术中心、工程研究中心和重点实验室，促进创新要素向企业集聚。提升国家机床质检中心、锂电检验检测中心的服务能力，争取更多标准化委员会落户枣庄，让更多“枣庄标准”引领行业发展。高水平办好枣庄创业大学，每个区（市）建立一所创业学院。推进各类创业示范园区建设，对获得省、市认定的大学生创业园给予扶持奖励。

　　三是激活创新创业主体。设立天使投资引导基金，用好市创业投资子基金、创业担保贷款和创业促就业专项资金，采取岗位开发补贴、跟进投资等方式，加大对创新创业主体的支持力度。深化工商注册登记制度改革，降低创业门槛，支持科技人才、草根能人、返乡务工人员创业，壮大市场主体规模，加快民营经济发展。深入实施大学生创业引领计划，扶持1000名大学生创业。积极开展创新创业训练营、创业大赛等活动，让勇于创新创业成为一种城市品格。

　　（五）突出抓好城乡统筹。坚持以人为本、以城带乡、以乡促城、城乡一体，推动城乡协调发展。

　　一是扎实开展新型城镇化八大行动。大力实施人口集聚、空间优化、产城融合、设施建设、环境提升、服务均等、文化传承、精细管理等八大行动，提高城镇化质量。稳步推进“镇改街”“村改居”，户籍人口城镇化率提高到45%。用足用活国家棚改政策，加快回迁安置房建设，棚改货币化安置率达到50%以上。按照“去存量、提质量”的思路，鼓励房地产开发企业适当降低商品住房价格、提升住房品质，打通保障房与商品房的通道，化解房地产库存。突出城乡结合部、老旧小区、集贸市场等重点部位，深入开展卫生综合整治，确保顺利通过国家卫生城市技术评估和暗访。加强非遗保护，实施“乡村记忆”工程，塑造城市文化品牌。突出薄弱环节，强化老城建设管理，加快实施基础设施改造十大工程，推动十电运煤专线迁建，优化市南工业区基础条件；重点实施北部城区棚改，推进老旧小区改造、背街小巷整治、城区亮化和环城绿化，完善城市功能，提升服务水平。

　　二是做优做靓大新城。依托大新城“七山八水”资源优势，推进“六大工程”建设，提升承载能力和城市品位。实施道路畅通工程，建成黄河路东路、复元三路南路和长江路东路，开工长白山路南延、民生路南延、厦门路提升等项目，启动世纪大道、大连路项目，提升城区道路通达能力。实施管廊建设工程，启动金沙江路地下综合管廊建设，用好地下空间资源，打造样板示范工程。实施山体增绿工程，搞好新城周边山体绿化保护，强化设施配套，建设城市山体森林公园。实施碧水绕城工程，依托南水北调工程，串连起黑峪水库、蟠龙河、凤鸣湖、龙潭水库、宏图河等水体，构建环城水系生态长廊。实施污水垃圾无害化处理工程，开工建设高新区污水处理厂，建成启用餐厨废弃物处理设施和生活垃圾焚烧发电项目，促进污水垃圾资源化利用。实施公共设施配套工程，抓好文体中心、实验高中三期、龙潭实验学校、职业中专二期、食品药品检验中心等项目建设，提升城市公共服务能力。

　　三是加强重大基础设施建设。完成北留线改建、枣曹线桥梁建设；加快推进万年闸复线船闸、枣庄港等在建工程；开工建设枣木高速东延、枣庄高铁换乘枢纽、枣济线及新城至滕州BRT、沂台线南延、京杭运河枣庄段二级航道整治等工程；做好枣庄机场、枣鱼高速、新台高速，以及枣庄至曲阜、临沂、徐州城际铁路等前期工作，加快形成四通八达、出行便利、带动发展的综合交通网络。抓好庄里水库及抽水蓄能电站规划建设，凤凰湖、何庄水库等南水北调配套工程建设，提高水资源保障能力。

　　（六）突出抓好生态建设。坚持“绿水青山就是金山银山”的理念，铁规治污、铁拳治污，增创绿色发展新优势。

　　一是在持续增绿上下功夫。启动国家生态园林城市创建，深入实施创森十大提升工程，新增造林面积9万亩、建成区绿化面积290公顷。以环中心城区绿道为框架，市场化建设运营好休闲驿站、森林人家、观光体验园等节点，争创国家生态公园。编制实施生态绿色廊道建设规划，新建提升绿色廊道300公里。大力倡导义务植树、社会捐绿，动员全民力量为枣庄增绿添彩。

　　二是在巩固治水上下功夫。健全寻源治污机制，严格南水北调“枣庄标准”，强化环境执法，倒逼企业治污减排，持续推进城区河道污水直排口整治，确保出境河流水质不反弹。加快用水方式转变，淘汰高耗水落后工艺设备，推广农业节水灌溉技术，严查违规凿井取水行为。完成农村集中式饮用水水源保护区划定，加强周村、羊庄等水源地保护，全面拆除一级水源保护区内畜禽养殖场、工业企业等污染源。强化饮用水水质监测，确保群众饮水安全。

　　三是在大气治理上下功夫。以PM2.5和PM10治理为突破口，最大程度减少大气污染源，消除群众的“心肺之患”，让群众少带口罩、少些“霾怨”。强化工业污染治理，加快煤电、水泥、化工、玻璃、砖瓦等企业提标改造，实施10蒸吨以上燃煤锅炉治理。强化扬尘治理，落实工地绿色作业方式，加大道路机械化清扫和洒水降尘力度。强化机动车尾气治理，加快老旧汽车淘汰，推进公交系统新能源汽车替换，倡导绿色出行。强化面源污染治理，全面推行餐饮行业安装高效油烟净化设施，依法取缔露天烧烤，狠抓秸秆禁烧和综合利用。

　　四是在生态修复上下功夫。夯实矿产资源监管属地责任，建立部门联合执法机制，严厉打击非法开采矿产资源行为。抓好破损山体、采矿塌陷地治理，不断提高生态自我修复能力。湿地保护率达到70%以上。抓好土壤环境状况详查和质量评估，实施土地分级分类管理。普及农业生产科技，严控农药使用，开展富营养化和酸化耕地修复试点，让“精疲力尽”的耕地休养生息。

　　（七）突出抓好民生实事。顺应群众期盼，办好惠民工程，全力发展社会事业，让群众拥有更多获得感。

　　一是打好脱贫攻坚战。落实精准扶贫、精准脱贫政策，再减少贫困人口8万人。对有劳动能力的，实行产业开发和转移就业扶贫；对“一方水土养不起一方人”的，实行易地搬迁安置脱贫；对居住在生态功能区的，实行生态补偿脱贫；对因病致贫的，提供医疗救助保障；对丧失劳动能力的，实行兜底性保障政策。

　　二是织密民生保障网。实施全民参保计划，努力实现人人享有社保。城乡低保标准分别提高到每人每月440元、每人每年3400元，五保集中、分散供养标准分别提高到每人每年4600元和3500元。新建保障性安居工程1万套以上，改造老旧小区40个、农村危房1000户。加快推进“气化枣庄”工程，实现镇镇通天然气。新增城市供热面积270万平方米。推动社会救助体系全覆盖。实施就业创业能力提升计划，城镇新增就业4.2万人，农村劳动力转移就业5万人。

　　三是提升公共服务水平。促进义务教育均衡发展，办好学前教育，鼓励发展民办教育，实施95所中小学新建、改扩建工程，完成129个“全面改薄”项目，逐步解决“大班额”问题；支持高等院校特色化办学。深化医药卫生体制改革，全面启动公立医院综合改革，推动分级诊疗，提升中医药服务能力。积极发展体育产业，办好各类体育赛事活动。推进基层文化服务中心建设。开展农村环境提升行动，实施村内路灯、道路、排水设施改造，高效运转环卫一体化设施。加快全域旅游厕所革命示范市建设，同步推进农村厕所改造。加强物业市场监管，坚决清退一批不合格物业企业，提升物业管理服务水平。抓好农村新型社区建设，新建改建农村社区服务中心50个。新建社区老年人日间照料中心15处、农村幸福院30处。新建改造农村公路320公里。落实全面两孩生育政策，促进人口均衡发展。

　　四是筑牢安全发展基石。推行网格化社会管理，完善矛盾纠纷多元化解机制。构建社会化治安防控体系，严厉打击各类违法犯罪活动。坚持党政同责、一岗双责、失职追责，强化企业主体责任，加大隐患排查整治力度，提高依法监管水平，确保安全生产形势持续稳定。开展“守护舌尖安全”专项整治，依法惩处制售假冒伪劣食品药品行为。持续开展双拥共建，积极配合军队改革。推动民族宗教、外事侨务、知识产权、档案史志、妇女儿童、红十字、老龄、残联、人防、地震、气象等事业全面发展。

四、进一步加强政府自身建设

　　办好枣庄的事情，主要看担当，关键在实干。要秉持“干在当下、成在实处”的理念，全面加强政府治理能力建设。

　　（一）加快建设责任政府。严格落实政府权力清单和部门责任清单，明晰政府部门干什么、群众有事该找谁，决不允许政府工作人员当“甩手掌柜”和“二传手”。坚持说了算、定了干，有的放矢、慎终如初，决不搞“东一榔头、西一棒槌”，不图虚名、不放“空炮”。健全绩效评估和问责制度，引入第三方评估，真督实查，打通关节，疏通堵点。强化效能监察，改进政风行风，坚决纠正慢作为、整治不作为、查处乱作为。

　　（二）加快建设法治政府。落实地方立法权改革精神，做好城乡建设管理、环境保护等方面的地方性法规起草工作。严格执行市政府工作规则和重大决策程序规定，建立重大行政决策终身追责制，完善政府法律顾问制度，探索政府新型智库建设，提高依法决策、科学决策水平。加强和改进行政复议、行政应诉工作，常态化开展普法宣传教育，提高政府工作人员法治意识和法治能力，严厉查处违法执法、粗暴执法行为。认真办理人大代表建议和政协提案，自觉接受人大法律监督、政协民主监督和社会舆论监督。

　　（三）加快建设创新政府。创新社会治理体系，整合政府系统信息资源，搭建政务云平台，扩大电子政务服务领域，推动政府数据资源向社会开放。创新公共服务供给方式，围绕医疗卫生、住房保障、社会救助、法律援助、公务出行等领域，加大政府购买服务力度。创新政务公开方式，完善政府信息发布制度，利用官方微信、微博、客户端等媒体，讲好“枣庄故事”，传递“枣庄声音”，提振“枣庄信心”。

　　（四）加快建设服务政府。巩固群众路线教育实践活动成果，健全领导干部联系群众、帮扶企业制度，真正做到干群零距离、服务零障碍、办事零延误。分类推进事业单位改革，深化重点领域价格改革。全面梳理公共服务事项，坚决砍掉各类无谓的证明和繁琐的手续，让群众办事更方便、更省心。开展市政府部门向市民报告、听市民意见、请市民评议、让市民监督活动，拓宽社情民意表达渠道。健全政府、企业、个人征信系统，完善社会信用体系，建设“诚信枣庄”，让守信者处处受益，让失信者寸步难行。

　　（五）加快建设廉洁政府。强化党风廉政建设主体责任，认真履行“一岗双责”，筑牢拒腐防变的“防火墙”。严格落实中央“八项规定”精神，自觉践行“三严三实”要求，扎紧作风建设的“篱笆墙”。牢固树立过紧日子的思想，严控“三公”经费支出。完成党政机关公务用车制度改革。推进公共资源交易集中管理，规范公共资源交易行为。推动审计监督全覆盖，对纳入预算的公共资金使用和重大投资项目、重点工程，实行全过程监督，让权力在“探照灯”和“摄像头”下运行。狠抓正风肃纪，坚决惩治腐败，进一步塑造干净干事的政府形象。

　　各位代表！回首“十二五”，艰辛奋斗铸辉煌；展望“十三五”，光明新篇待开启。让我们紧密团结在以习近平同志为总书记的党中央周围，在市委的坚强领导下，携起手来干事业，握紧拳头抓发展，为提前全面建成小康社会而努力奋斗！
